# Early Fish Myoseptal Cells: Insights from the Trout and Relationships with Amniote Axial Tenocytes

**DOI:** 10.1371/journal.pone.0091876

**Published:** 2014-03-12

**Authors:** Yoann Bricard, Cécile Rallière, Veronique Lebret, Florence Lefevre, Pierre-Yves Rescan

**Affiliations:** French National Institute for Agricultural Research, Fish Physiology and Genomics, Rennes, France; UMR CNRS 5242 - ENS de Lyon- Université Lyon 1, France

## Abstract

The trunk muscle in fish is organized as longitudinal series of myomeres which are separated by sheets of connective tissue called myoseptum to which myofibers attach. In this study we show in the trout that the myoseptum separating two somites is initially acellular and composed of matricial components such as fibronectin, laminin and collagen I. However, myoseptal cells forming a continuum with skeletogenic cells surrounding axial structures are observed between adjacent myotomes after the completion of somitogenesis. The myoseptal cells do not express myogenic markers such as Pax3, Pax7 and myogenin but express several tendon-associated collagens including *col1a1*, *col5a2* and *col12a1* and angiopoietin-like 7, which is a secreted molecule involved in matrix remodelling. Using *col1a1* as a marker gene, we observed in developing trout embryo an initial labelling in disseminating cells ventral to the myotome. Later, labelled cells were found more dorsally encircling the notochord or invading the intermyotomal space. This opens the possibility that the sclerotome gives rise not only to skeletogenic mesenchymal cells, as previously reported, but also to myoseptal cells. We furthermore show that myoseptal cells differ from skeletogenic cells found around the notochord by the specific expression of Scleraxis, a distinctive marker of tendon cells in amniotes. In conclusion, the location, the molecular signature and the possible sclerotomal origin of the myoseptal cells suggest that the fish myoseptal cells are homologous to the axial tenocytes in amniotes.

## Introduction

The musculoskeletal system is a multicomponent system composed of muscles, bones and connective tissues. In fish, the musculoskeletal system is relatively simple: a connective tissue called myoseptum separates W-shaped myomeres that are arranged in a longitudinal series [1]. Most fish species also have a horizontal septum that divides the myotomal muscle into epaxial and hypaxial domains. The myoseptum is medially inserted on the bony axial skeleton and is laterally connected to the collagenous dermis. Both myoseptum in fish and tendon in amniotes serve as transmitters of muscle contractility to bones, and it has been suggested that these two structures are functionally homologous [2], [3]. As in amniote tendon, the fish myoseptum is contiguous with the perimysium that surrounds a group of muscle fibres and with the endomysium that surrounds each individual muscle fibre [4].

Muscle differentiation and development are well documented in fish. Fish axial skeletal muscles contain two major fibre types: the superficial slow muscle fibres and the deep fast muscle fibres. Single-cell labelling experiments have shown that the somite adaxial cells, initially next to the notochord, migrate radially to form the embryonic slow muscle fibres at the lateral surface of the myotome, underneath the dermomyotome-like epithelium. Cells of the posterior somitic compartment differentiate into fast muscle fibres, whereas those of the anterior somitic compartment ultimately form the superficial dermomyotome-like epithelium. This epithelium provides myogenic precursor cells necessary for myotome growth (for review see [5] [6]).

Most studies on the formation of the fish myoseptum focused on the myotendinous junction development and the deposition of myoseptal matrix that follow somite formation. Using the zebrafish model, Henry and collaborators have shown that the morphogenesis of the nascent myotendinous junction is associated with an enrichment of extracellular matrix/focal adhesion/dystroglycan complex components at the myotome boundary, which limits myofibre elongation [7]. Among the extracellular matrix (ECM) molecules that are deposited in the developing myoseptal matrix of the zebrafish are found laminin and fibronectin [7], [8], tenascin [9], collagen XII [10] and collagen XXII [11]. It has been shown in zebrafish that fibronectin at the myotendinous junction is down-regulated medial to migrating slow–twitch muscle fibres whereas laminin level remains constant [8]. This suggests that dynamic changes in the molecular composition of the extracellular matrix separating adjacent myotomes not only impact the biomechanical properties of the myosepta but may also mediate normal musculoskeletal development.

Using electron microscopy examinations, Kudo et al. reported that the myoseptum of 48 hpf zebrafish embryos consists of fine cytoplasmic processes from a few cells in the triangular region surrounded by adjacent somites and notochord or spinal cord [12]. Additionally, Charvet et al. reported the presence of fibroblast-like cells in the developing myoseptum of late (6 dpf) zebrafish embryos [13]. Thus, some cellular structures become apparent in the intersomitic space of the late fish embryo, but they are poorly characterized. The purpose of this study is to present some aspects of the myoseptal cells that invade the intermyotomal space in late fish embryo with emphasis on their molecular signature. Trout was chosen for this study because the organ rudiments in this fish species are larger, and early stage development is slower than in fish model species such as zebrafish or medaka. Overall, our findings lead us to propose that the fish myoseptal cells that invade the intermyotomal space at the completion of somitogenesis show characteristics of amniote axial tenocytes.

## Materials and Methods

### Ethics statement

This work used early trout embryos. All experiments performed in this study followed the recommendations of the “Comité National de Reflexion Ethique sur l'Experimentation Animale” of the Ministry of Higher Education and Research and were approved by the Local Animal Care and Use Committee (approval n° 7I12).

### Semi-thin section preparation

The specimens were immersed for 2 h at 4°C in 2.5% glutaraldehyde diluted in 0.1 M phosphate buffer (pH 7.4). They were subsequently postfixed in a mixture of 1% osmium tetraoxide for 1 h, dehydrated by passage through ascending concentrations of ethanol, and embedded in epoxy resin. Semi-thin sections of these specimens were cut on a Reichert Supernova ultramicrotome.

### Whole-mount in situ hybridisation

*Col1a1* chain (GenBank accession number BX086928), *col5a2* chain (GenBank accession number CA384576), *col12a1* chain (GenBank accession number BX077257), angiopoietin like-7 (GenBank accession number KF640214), and osteoblast-specific factor2/periostin (GenBank accession number KF640216) cDNAs have been identified from a large-scale rainbow trout 3′ and 5′ sequencing project [14]. Scleraxis cDNA (Genbank accession number KF640215) was amplified from a rainbow trout embryonic cDNA library using primers designed from a genomic contig encompassing scleraxis exons 1 and 2. Digoxigenin-labelled antisense RNA probes were synthesised from a PCR-amplified template using an appropriate RNA polymerase. The embryos were dechorionated with fine forceps and fixed overnight at 4°C in paraformaldehyde in PBS. Specimens were dehydrated and stored in methanol at −20°C. Following rehydration in graded methanol/PBS baths, embryos were processed according to established procedures [15] with minor modifications. Depending on the embryonic stage, different times, temperatures, and concentrations were chosen for proteinase K treatment.

### In situ hybridisation of embryo sections

For late embryos (eyed stage onwards), in situ hybridisations were performed on sections of rainbow trout embryos. Embryos were embedded using the cryowax protocol as detailed by Duran et al. [16]. Frontal and transverse sections were then cut, mounted and dewaxed [16]. In situ hybridisation procedures were performed according to Gabillard et al. [17].

### Immunohistochemistry

Dewaxed sections were washed in phosphate buffered saline buffer, incubated in 1% BSA in TBS for 10 minutes before a further overnight incubation in primary antibody diluted in TBS. After washing, sections were incubated in biotin-conjugated or Alexa Fluor-conjugated secondary antibodies diluted in TBS and washed again in TBS. When biotinylated secondary antibodies were used, the sections were incubated with streptavidin peroxidase, and the staining reaction was performed using diaminobenzidine/H_2_O_2_. The following antibodies were used: polyclonal anti-phospho-histone H3 (1∶200; Millipore, catalogue number 06-570), polyclonal anti-Laminin (1∶200; Sigma, catalogue number L-9393), polyclonal anti-fibronectin (1∶200; Sigma, catalogue number F-3648) and polyclonal anti-salmon fish type I collagen (1∶1000; Novotec, catalogue number S20171). The secondary antibodies used were biotinylated goat anti-rabbit antibodies (Thermo scientific) and Alexa Fluor 488-conjugated goat anti-rabbit antibodies (1∶200; Invitrogen).

## Results

### Early concentration of ECM components at the boundaries of somites

**Figure 1:**
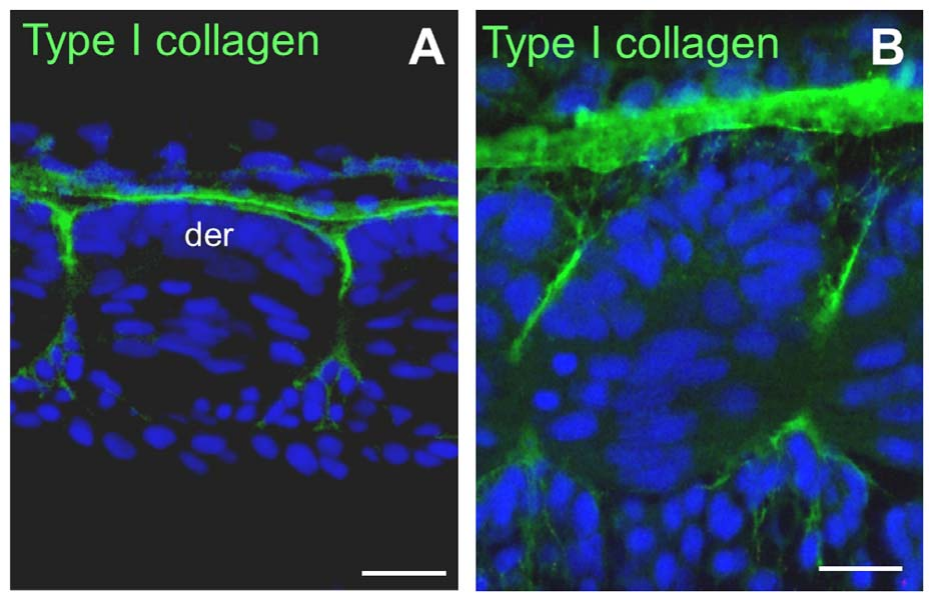
Collagen I is concentrated at the surface of the somites. (**A and B**) Frontal sections through the trunk of a 13 dpf trout embryo. (**A**) Posterior tail. Collagen I immunofluorescence localises to the anterior and posterior edges and at the lateral surface of the dermomyotome. (**B**) Anterior tail. Collagen I immunofluorescence is present along the space separating adjacent somites. der: dermomyotome. Scale bars in A and B, 15 μm.

Somites are generated repeatedly from the presomitic mesoderm in an anterior to posterior progression. Frontal sections through the trunk region of the trout embryo showed that the area between two adjacent newly formed somites was acellular. Immunolocalisation of extracellular matrix components revealed immunoreactivity for laminin and fibronectin at the boundaries of somites as they formed (Figure S1). Later on, immunoreactivity for collagen I was also observed, initially in the vicinity of the rostral and caudal edges of the dermomyotome and then along the space separating two somites (Fig. 1A and B). These data show that acellular connective tissue is initially present at the surface of the somites.

### The myoseptal stroma is progressively invaded by mesenchymal cells

**Figure 2:**
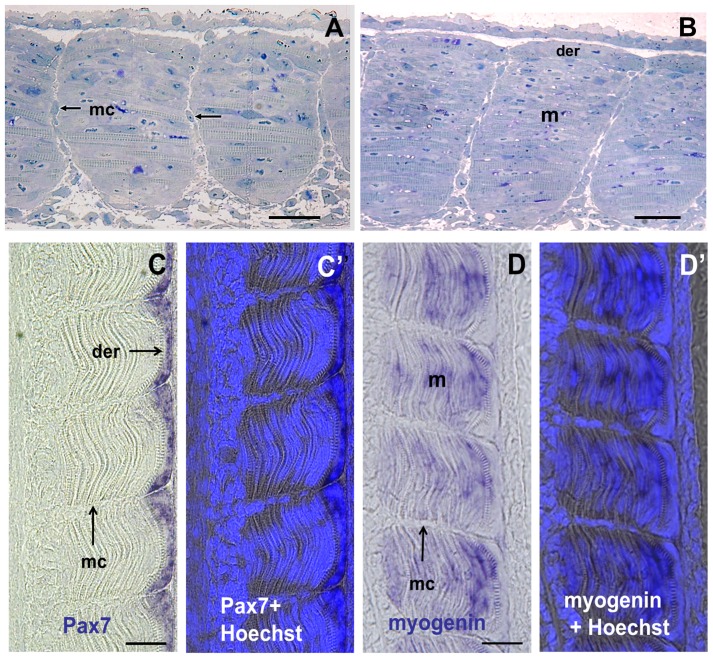
The intermyotomal space is colonized by fibroblast-like cells that do not express myogenic markers. (**A and B**) Semithin frontal sections through the trunk of an eyed stage (17 dpf) embryo with final number of somites and pigmented eyes. (**A**) Posterior tail. Myoseptal cells are visible in the medial half (arrows) of the space separating two somites. (**B**) Anterior tail. Myoseptal cells are visible throughout the medio-lateral extent of the intermyotomal space. (**C**) Pax 7 expression is restricted to cells forming the dermomyotome-like epithelium at the surface of the myotome. (**C′**) Merged image showing Pax7 labeling and Hoechst staining for nuclei visualisation. (**D**) Myogenin expression is observed in the primary myotome located below the dermomyotome-like (**D′**) Merged image showing myogenin labeling and Hoechst staining. mc: myoseptal cells, m: myotome, der: dermomyotome. Scale bars in A, B, C and D, 25 μm.

A remarkable change occurred in the organisation of the myoseptal stroma after the completion of somitogenesis (15 dpf) that takes place well before hatching (30 dpf). From this stage onwards, fibroblast-like myoseptal cells were observed in the intersomitic space, initially in the medial aspect of the myoseptal matrix and then more laterally as somites mature along the anteroposterior axis (Fig. 2A and B). Only rare myoseptal cells (<1%) were found to be phospho-histone H3 (H3P) positive at the eyed stage (17dpf) (figure S2). This indicated that only a small fraction of myoseptal cells were in S-Phase, suggesting that the majority of myoseptal cells were non-dividing as they invaded the intermyotomal space.

### Identification of genes expressed in myoseptal cells

**Figure 3:**
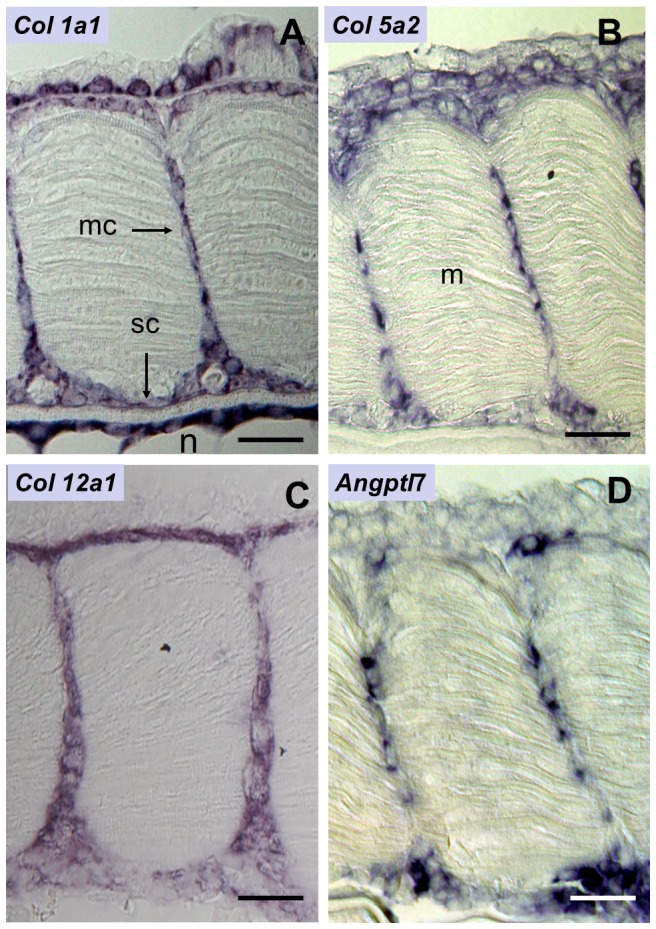
Myoseptal cells express genes involved in extracellular matrix production and remodelling. (**A**–**D**) Frontal sections of eyed stage (17 dpf) embryos. Expression of (**A**) *col1a1*, (**B**) *col5a2*, (**C**) *col12a1* and (**D**) Angiopoietin-7 like. mc: myoseptal cell; sc: skeletogenic cells; m: myotome; n: notochord. Scale bars, 20 μm.

We used *in situ* hybridisation to identify genes expressed in the fibroblast-like myoseptal cells. We first examined whether the myoseptal cells expressed myogenic markers, such as Pax3, Pax7 and myogenin. We found that myoseptal cells did not express these markers (Fig. 2C–D and C′–D′) and therefore, were not myogenic. In contrast, Pax3 (not shown) and Pax7 (Fig. 2C and C′) were expressed in the myogenic progenitors forming the dermomyotome-like epithelium at the surface of the primary myotome while myogenin was expressed in the differentiating primary myotome (Fig 2D and D′). We further examined the expression of matricial compounds such as collagens. The full-length cDNA sequence of trout *col1a1* has been previously described [18]. High-throughput sequencing of the trout genome led us to further identify the full-length cDNA sequences of trout *col5a2* and *col12a1*, the latter of which is classified as a FACIT (Fibril Associated Collagen with Interrupted Triple helix). Using in situ hybridisation with riboprobes complementary to these cDNAs, we observed that *col1a1*, *col5a2* and *col12a1* were expressed in the myoseptal cells located between adjacent myomeres (Fig. 3A–C). We also found that medial myoseptal cells were in close contact with collagen expressing skeletogenic cells at the surface of the axial structures. Thus, myoseptal cells and skeletogenic cells formed a continuum connecting myotomes to the developing axial skeleton. Taking advantage of a random in situ hybridisation screen (Rallière and Rescan, unpublished data), we found that myoseptal cells expressed angiopoietin-like 7 (Fig. 3D; for amino-acid sequence of angiopoietin-like 7 see Figure S3), a secreted glycoprotein involved in extracellular matrix remodelling [19]. Collectively, our data support the view that myoseptal cells are involved in the formation and maturation of the extracellular matrix separating adjacent myomeres. In course of this study we observed that *col1a1*, *col5a2* and *col12a1* [20] were expressed by the dermomyotome-like epithelium found at the surface of the primary myotome, showing that this somite-derived compartment provides a major contribution to extracellular matrix production (Figure S4).

### The apparent movement of *col1a1* expressing cells in developing trout embryo opens the possibility that sclerotome gives rise to myoseptal cells

**Figure 4:**
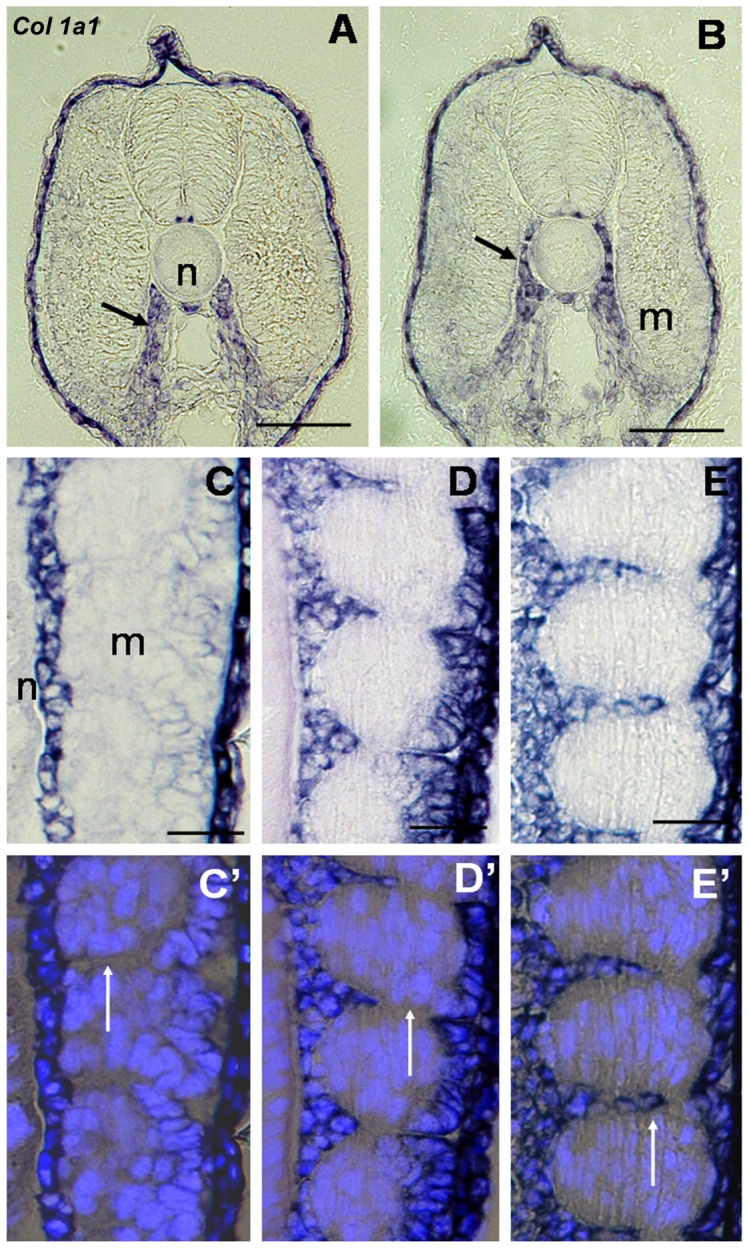
Apparent movement of *col1a1* expressing cells suggests a sclerotomal origin of myoseptal cells. (**A**) Transverse section of a 13 dpf embryo. *Col1a1* staining is present in the ventrally located sclerotome cells (arrow). (**B**) Transverse section of a 14 dpf embryon. Labelled cells have migrated dorsally to surround the notochord (arrow). (**C**–**E**) Frontal section of trout embryo at the level of the notochord. (**C**) 14 dpf embryo. Labelled cells surround the notochord. (**D**) 15 dpf embryo. Some labelled cells occupy the medial aspect of the intermyotomal space. (**E**) 16 dpf embryo. The medial-lateral extent of the intermyotomal space contains labeled cells. (**C**′–**E**′) Merged images showing *col1a1* labelling and Hoechst nuclear staining, white arrows indicate the intermyotomal space. n: notochord; m: myotome. Scale bars in A and B, 50 μm; C, C′, D, D′, E and E′, 30 μm.

To provide preliminary insight into the origin of the myoseptal fibroblasts, we examined the expression of the myoseptal cell marker *col1a1* during trout development. Transverse sections through trout embryos revealed an early labelling of *col1a1* in disseminating cells ventral to the myotome (Fig. 4A). These cells presumably originate from the sclerotome. Later on, labelled cells were found more dorsally along a path between the notochord and the myotome as expected for sclerotome-derived skeletogenic cells contributing to the formation of the axial skeleton (Fig. 4B). However, frontal sections through trout embryos showed that not all cells expressing *col1a1* surrounded the axial structure (Fig. 4C); some of them, instead, were found to colonise, in an apparent medio-lateral progression, the space separating two adjacent myotomes (Fig. 4D–E and D′–E′). Thus, the apparent movement of collagen I expressing cells is compatible with the view that the fish sclerotome gives rise not only to cells contributing to the formation of the axial skeleton as previously reported [21], but also to cells that intercalate between myotomes to form the myoseptum.

### Myoseptal cells specifically express Scleraxis

**Figure 5:**
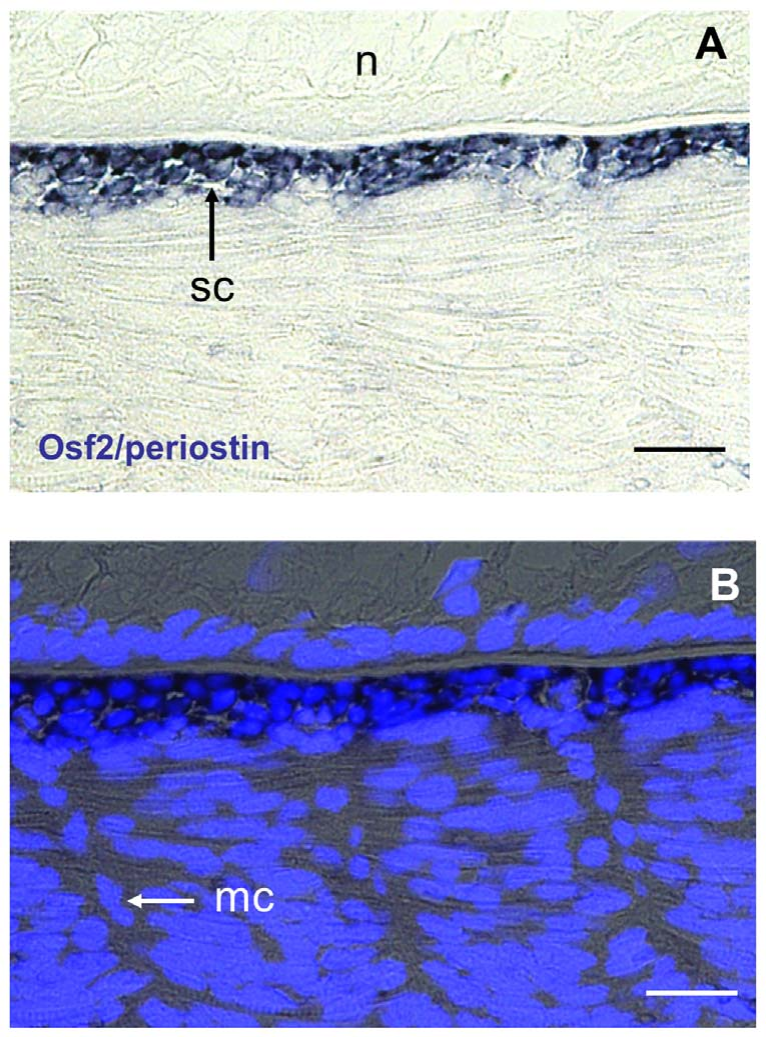
Skeletogenic cell specifically express osteoblast-specific factor2/periostin. Eyed-stage (17dpf) embryo, frontal section. (**A**) Labelling is restricted to sclerotome-derived skeletogenic cells (sc) surrounding the notochord. (**B**) Merged images with nuclei stained with Hoechst to visualise myoseptal cells (ms). Scale bars in A and B, 20 μm.

**Figure 6:**
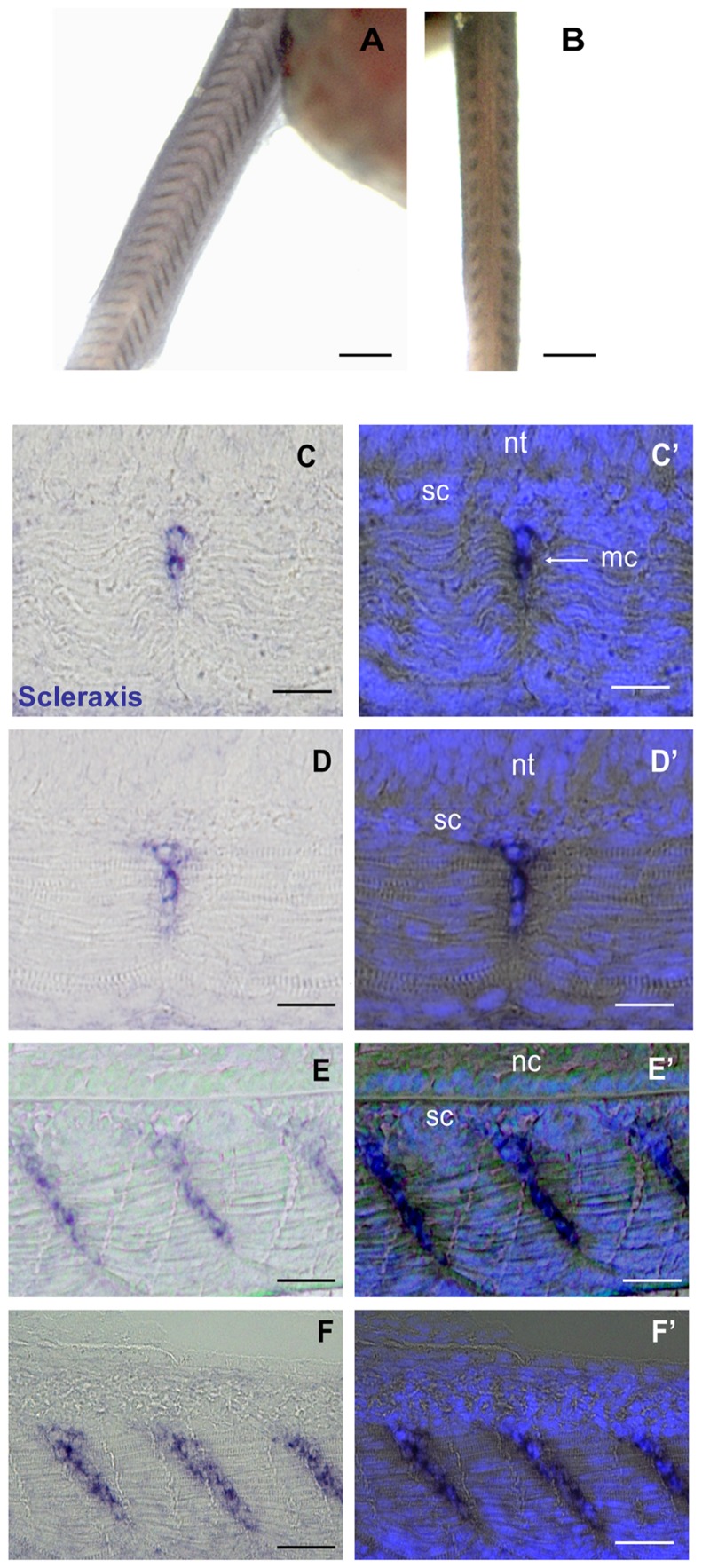
Expression of Scleraxis in myoseptal cells. Eyed stage (17dpf) embryo. (**A**–**B**) whole mount in situ hybridization. (**A**) Lateral view. (**B**) Dorsal view. Scleraxis is localised at the anterior and posterior myotome borders. (**C**–**F**) Serial frontal sections through the trunk of an eyed stage trout embryo. (**C**) A section at the dorsal neural tube level shows few labelled myoseptal cells medially in the intermyotomal space. (**D**) A section at the ventral neural tube level shows that labelled myoseptal cells are visible within the medial half of the intermyotomal space. (**E**) Sections at the level of the notochord and (**F**) ventral to the notochord show that labelled cells are present throughout the medio-lateral extent of the intermyotomal space. (**C**′–**F**′) Merged images showing Scleraxis labelling and Hoechst nuclear staining; there is no labelling in skeletogenic cells immediately adjacent to the neural tube and notochord. nt: neural tube; n: notochord, mc: myoseptal cell; sc: skeletogenic cells. Scale bars in A, 300 μm; B, 200 μm Scale bars in C, C′, D and D′, 25 μm; E, E′, F and F′, 40 μm.

To further differentiate myoseptal cells in the intermyotomal space from mesenchymal cells surrounding the notochord, we examined the expression of scleraxis, a bHLH transcriptional regulator that, in amniotes, marks specifically sclerotome-derived cells fated to form tendons and ligaments in amniotes [22]) and osteoblast-specific factor2/periostin, a matricellular protein notably found at the surface of the periosteum lining the outer surface of bones [23]. By RT-PCR, we generated a full-length cDNA encoding a trout scleraxis orthologue (Figure S5) that exhibited 78% identity with zebrafish scleraxis homolog A and 62% identity with zebrafish scleraxis homolog B. However, we identified a full length EST cDNA from a large-scale rainbow trout sequencing project [14] that encodes a trout osteoblast-specific factor2/periostin orthologue (Figure S6). In situ hybridisation with labelled riboprobes derived from these cDNAs showed that sclerotome-derived cells found around axial structures distinctly expressed osteoblast-specific factor2/periostin (Fig. 5A and B) while cells invading the intermyotomal space specifically expressed Scleraxis (Fig. 6). Interestingly, we observed that the apparent medio-lateral invasion of the myoseptal matrix by Scleraxis-positive myoseptal cells followed the encirclement of axial structures by skeletogenic cells (Fig. 6C–F and C′–F′). This indicates that the myoseptum and cartilage lineages are distinct in terms of gene expression and migration pattern. Unfortunately, scleraxis transcripts were undetectable in the early somites, which impeded further description of the emergence and migration pathways of myoseptal cell progenitors.

## Discussion

In this study, we describe some aspects of the formation of the myoseptum separating adjacent myomeres in trout embryos and provide evidences, mainly based on gene expression, suggesting that fish myoseptal cells are homologous of amniote axial tenocytes. We found that the myoseptum in the trout embryo is initially acellular and is composed of ECM compounds, such as laminin and fibronectin, as reported in zebrafish [7], [8], and collagen I. It has been shown that fibronectin knockdown in zebrafish disrupts somite boundary formation and muscle morphogenesis [24] and that loss of laminin α2 chain results in alteration of the myotendinous junction as observed in the zebrafish *candyfloss* (laminin α2) mutant [25]. Normal polymerisation of laminin at the myotendinous junction requires nicotinamide riboside kinase 2 b (Nrk2b) activity as revealed by the phenotype of Nrk2b-deficient zebrafish [26]. Our observation that collagen I is deposited in the myoseptal matrix extends previous studies showing the presence of collagen XII [10] and collagen XXII [11] in the intersomitic space. In this regard, it is interesting to note that collagen XII *in vitro* binds to fibrils of collagen I [27] suggesting specific interactions between these two molecules in the early myoseptal matrix.

The observation that myoseptum is initially acellular raises the question of the sources of the ECM molecules forming the myoseptal matrix. In zebrafish, fibronectin and laminin are expressed by the presomitic mesoderm and developing somites, respectively [28], [29]. An expression of *col22a1* concentrated to muscle cell extremities close to the vertical myosepta has also been reported in zebrafish embryos [11]. In trout, we confirm that collagen I, which is initially deposited at the caudal and rostral edges of the dermomyotome before being distributed throughout the lateral to the medial extent of the somite boundary, is expressed by the dermomyotome-like epithelium [20]. Collectively, these data indicate a major contribution of presomitic mesoderm cells or somite-derived cells in the early formation of the myoseptal matrix. However, it has been reported that ectopic secretion of laminin from different non-muscle tissues can localise to zones of muscle attachment and ameliorate the phenotype of zebrafish *candyfloss* (laminin α2) mutant [30].

A remarkable change in the organisation of the myoseptum occurs after the completion of somitogenesis. From this stage onwards, mesenchymal cells are observed in the intermyotomal space. Mesenchymal cells have also been reported in the myoseptum of zebrafish [13] and shi drum larvae [31], showing that cell colonisation of the myoseptal stroma is a general feature of myoseptum morphogenesis in fish. We further showed that myoseptal cells express fibrillar collagen such as *col1a1* and *col5a2* as well as FACIT such as *col12a1*. These observations suggest that myoseptal cells are likely to induce significant changes in composition, structure and biomechanical properties of the primary myoseptal matrix. For example, other studies have reported the formation of a dense network of collagen fibrils in zebrafish myoseptum that correlates with the invasion of fibroblasts [13].

A major question in developmental biology relates to the origin of cells forming the fish myoseptum. Using *col1a1* as a marker gene, we found that its expression was first detected in disseminating cells ventral to the myotome before being observed in presumptive skeletogenic cells found along a path between axial structures and the myotome, and cells invading the intermyotomal space. This apparent movement of *col1a1* expressing cells in early trout embryo opens the possibility that the sclerotome gives rise to a population of cells involved in the development of the axial skeleton (i.e., the formation of the neural and haemal arch rudiments) as previously reported [21], [32], as well as to a cell population that participates to the formation of the fish myoseptum. However, to definitely demonstrate that sclerotome in fish produces myoseptal cells it will be necessary to achieve genetic lineage labelling and time-lapse analysis [33].

We observed that myoseptal cells separating adjacent myotomes abut medially against skeletogenic cells surrounding the notochord. We further show in this study that myoseptal cells differ from the skeletogenic cells by gene expression: myoseptal cells expressed scleraxis, a distinctive marker of tenocytes in amniotes, while skeletogenic cells specifically expressed osteoblast-specific factor 2/periostin, a matricellular protein expressed *in vivo* and *in vitro* during mouse osteoblast differentiation [34], [35]. The trout scleraxis gene we identified exhibits higher identity with zebrafish *scleraxis homolog A* (*scxa*) than with zebrafish *scleraxis homolog B (scxb)*. Given the ancient whole-genome duplication that occurred in the teleost fish lineage, after split of lobe- and ray-finned lineage [36], it is possible that an additional scleraxis gene orthologous to zebrafish *scxb* exists in trout genome. Further studies are needed to elucidate this point. We were unable to detect scleraxis and osteoblast-specific factor 2/periostin transcripts in the early trout embryo, preventing further description of the early emergence of the myoseptum and cartilage lineages. In future studies it will be important to identify genes whose expression marks early progenitors of the myoseptum and cartilage lineages, and to precisely describe the migration pathway of myoseptal cell progenitors throughout the intermyotomal space.

**Figure 7:**
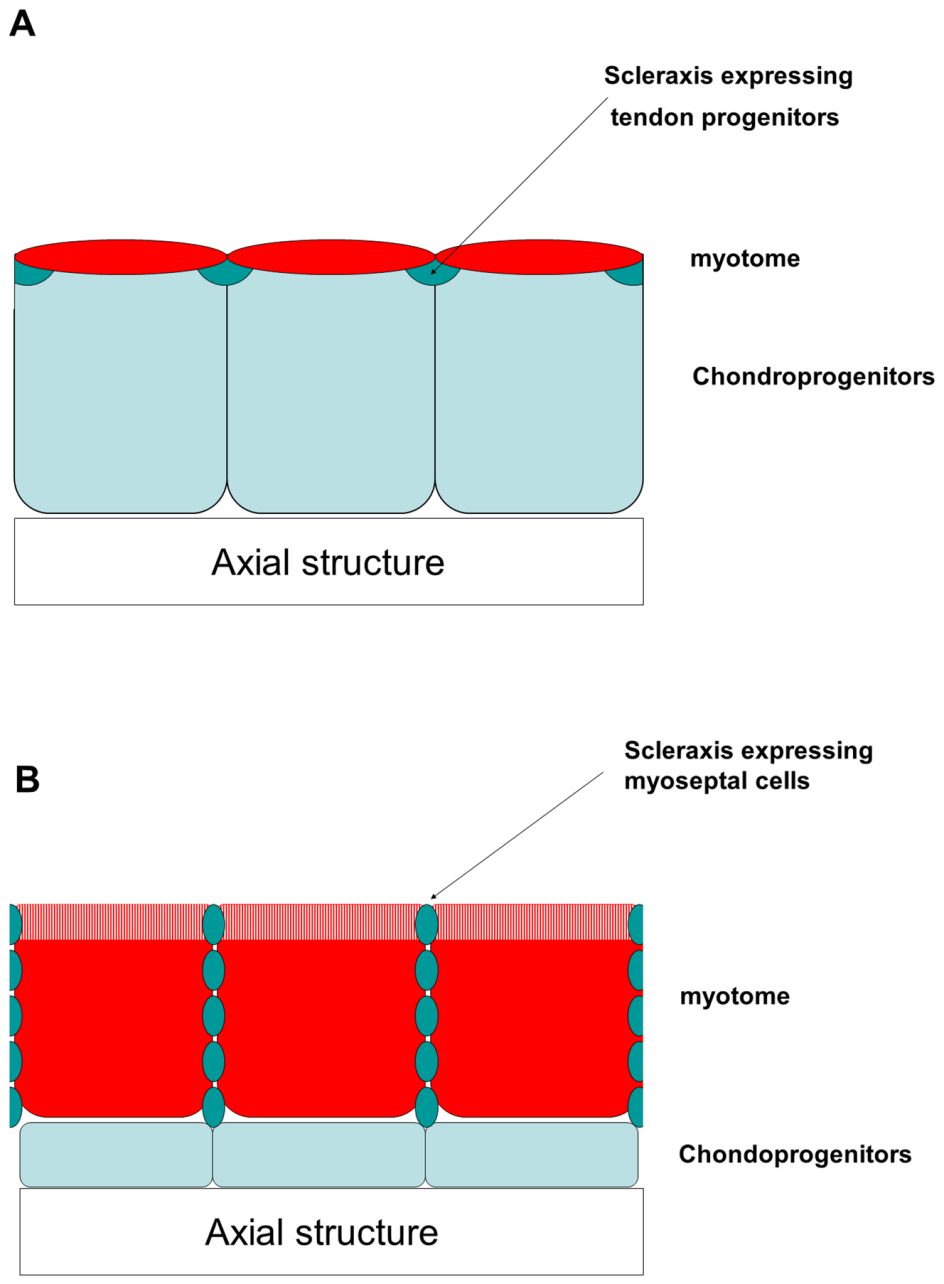
Comparison of the early axial musculoskeletal system between amniote and fish. Schematic frontal sections through trunk region of amniote (**A**) and fish embryos (**B**). (**A**) Amniote embryo. The sclerotome that derives from an important portion of the somite gives rise to chondroprogenitors surrounding axial structures (notochord and neural tube). Scleraxis expressing tendon progenitors originating from the dorsolateral edge of the early sclerotome lie between adjacent myotomes (adapted from [13]). (**B**) Fish embryo (17dpf). The sclerotome that represents a reduced somite derivative gives rise to a limited number of chondroprogenitors that express osteoblast-specific factor2/periostin. Adjacent myotomes are separated by Scleraxis-expressing myoseptal cells. A dermomyotome (white and red vertical lines) at the surface of the myotome persists late during fish development and expresses high levels of *col1a1*, *col5a2* and *col12a1*.

Fish myoseptum is generally regarded as tendon, both are connective tissues and both transmit the force generated by muscle contraction to the skeleton [2], [3]. We show in this study that fibroblaste-like cells myoseptal cells invade the myoseptal matrix after the completion of somitogenesis. These cells, that abut medially against skeletogenic cells, express tendon-associated collagens, such as collagens I, V and XII [37] and scleraxis, a highly specific marker of tenocytes in amniotes [22] and anurans [38]. Also myoseptal cells may derived from the sclerotome as shown by the apparent movement of collagen I expressing cells in the early embryo. Together, these features that are common to amniote axial tenocytes [22], [37] further support a homology between fish myoseptum and amniote tendons, specifically those tendons associated with the axial skeleton (Fig.7).

The external epithelium at the surface of the fish embryonic myotome has been shown to express Pax3 and Pax7 [39], [40] which are two markers of muscle precursors, and to provide myogenic precursor cells necessary for growth of the embryonic myotome [41], [42], [43]. In this study, we show that this structure also expresses *coll5a2* and *col12a1* as the dermis. This observation is in agreement with the view that the external epithelium is homologous to the amniote dermomyotome [39]. Whether this epithelium gives rise to dermis cells is still debated [41], [44]; however, it is now clear that this structure that expresses dermo-1 and *col1a1* [40], [20] produces a large set of ECM molecules and thus contributes to the formation of the primitive acellular connective tissue surrounding somites, especially in domains facing the epidermis.

## Conclusions

In this study we provide evidences that myoseptal cells in teleosts have the topological and molecular characteristics of amniote axial tenocytes suggesting that the fish myoseptum is homologous to the axial tendons in amniotes. Moreover, we show that the fish external cell layer surrounding the primary myotome expresses a large set of matricial proteins, further supporting the view that this structure is closely related to the dermomyotome of amniotes.

## Supporting Information

Figure S1 **Laminin and fibronectin localisation.** (A and B) Frontal sections through the trunk of a 9 dpf trout embryo. Laminin (**A**) and fibronectin (**B**) are concentrated at somite boundaries. Scale bars in A and B, 20 μm.
(TIF)

Figure S2 **Immunolocalisation of H3P in myoseptal cells in a frontal section through the trunk of a 21 dpf trout embryo.** Nuclei were stained with Hoechst. Labelling is observed in rare myoseptal cells (arrow). Scale bars, 30 μm.
(TIF)

Figure S3 **Comparison of the predicted trout and murine angiopoietin-like 7 protein sequence.** Shading indicates identity.
(TIF)

Figure S4 **Expression of** ***col5a2*** **(A) and** ***col12a1*** **(B) in a trout embryo at stage 13 dpf (somitogenesis is not completed).** Frontal sections. Collagen V and XII transcripts strongly accumulate in the dermomyotome-like epithelium (arrow) at the surface of the primary myotome (PM). Note that labelling for *col12a1* is still reduced in sclerotome-derived cells surrounding the notochord. Scale bars in A and B, 30 μm.
(TIF)

Figure S5 **Comparison of the predicted trout and murine scleraxis protein sequences.** Shading indicates identity. The highly conserved bHLH domain is marked with a bar.
(TIF)

Figure S6 **Comparison of the predicted trout and murine Osteoblast-specific factor2/Periostin protein sequence.** Shading indicates identity. Positions of the signal sequence (green segment), the cysteine-rich region (blue segment) and the four fasciclin I-like repeats (R1-4) (red segments) are indicated.
(TIF)
