# Supplementary figures and images for: Early Fish Myoseptal Cells: Insights from the Trout and Relationships with Amniote Axial Tenocytes
Source: PLoS One. 2014 Mar 12;9(3):e91876. doi: 10.1371/journal.pone.0091876 (PMC3951490; doi:10.1371/journal.pone.0091876)

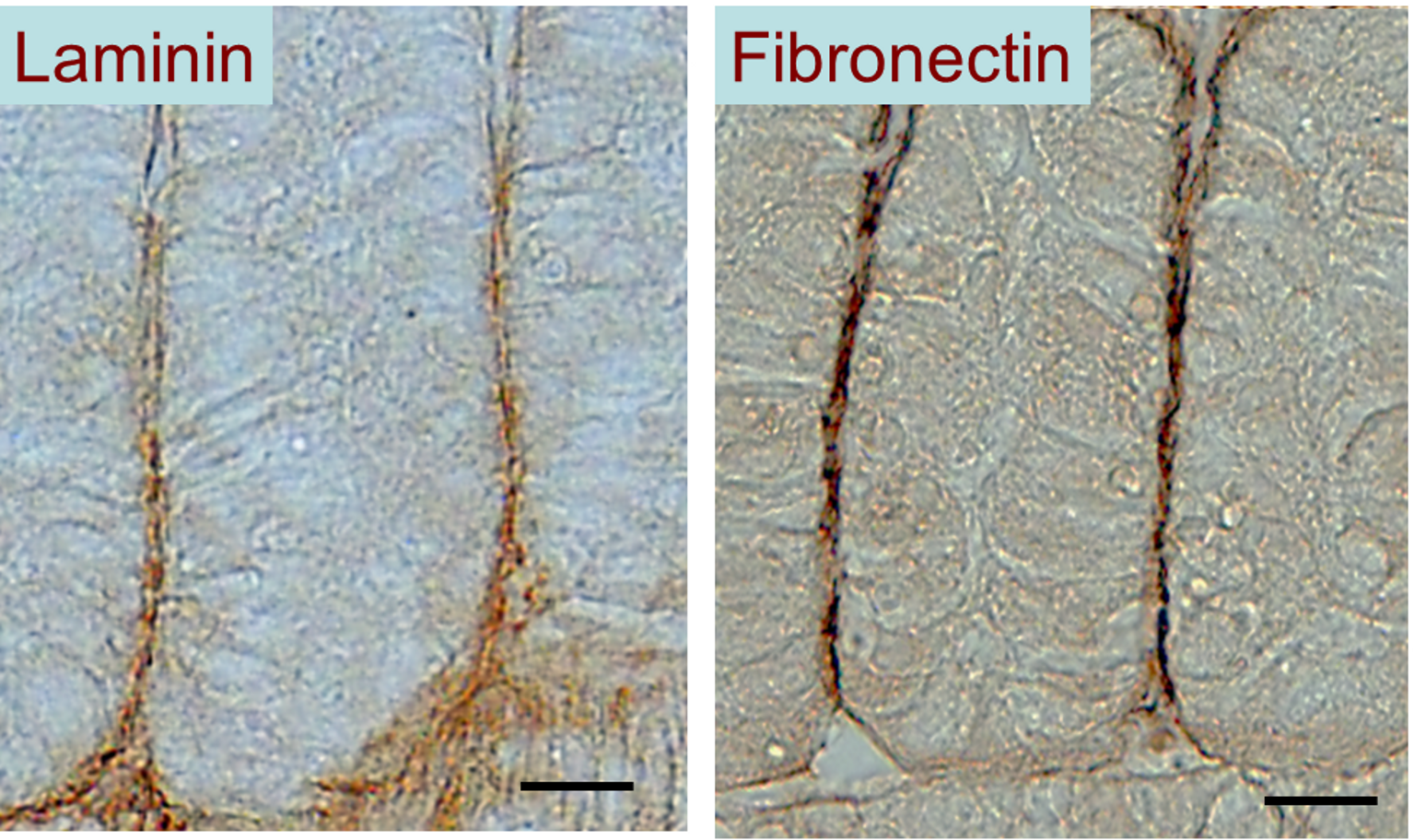

Supplement: Figure S1 — Laminin and fibronectin localisation. (A and B) Frontal sections through the trunk of a 9 dpf trout embryo. Laminin (A) and fibronectin (B) are concentrated at somite boundaries. Scale bars in A and B, 20 μm. (TIF) [file pone.0091876.s001.tif]

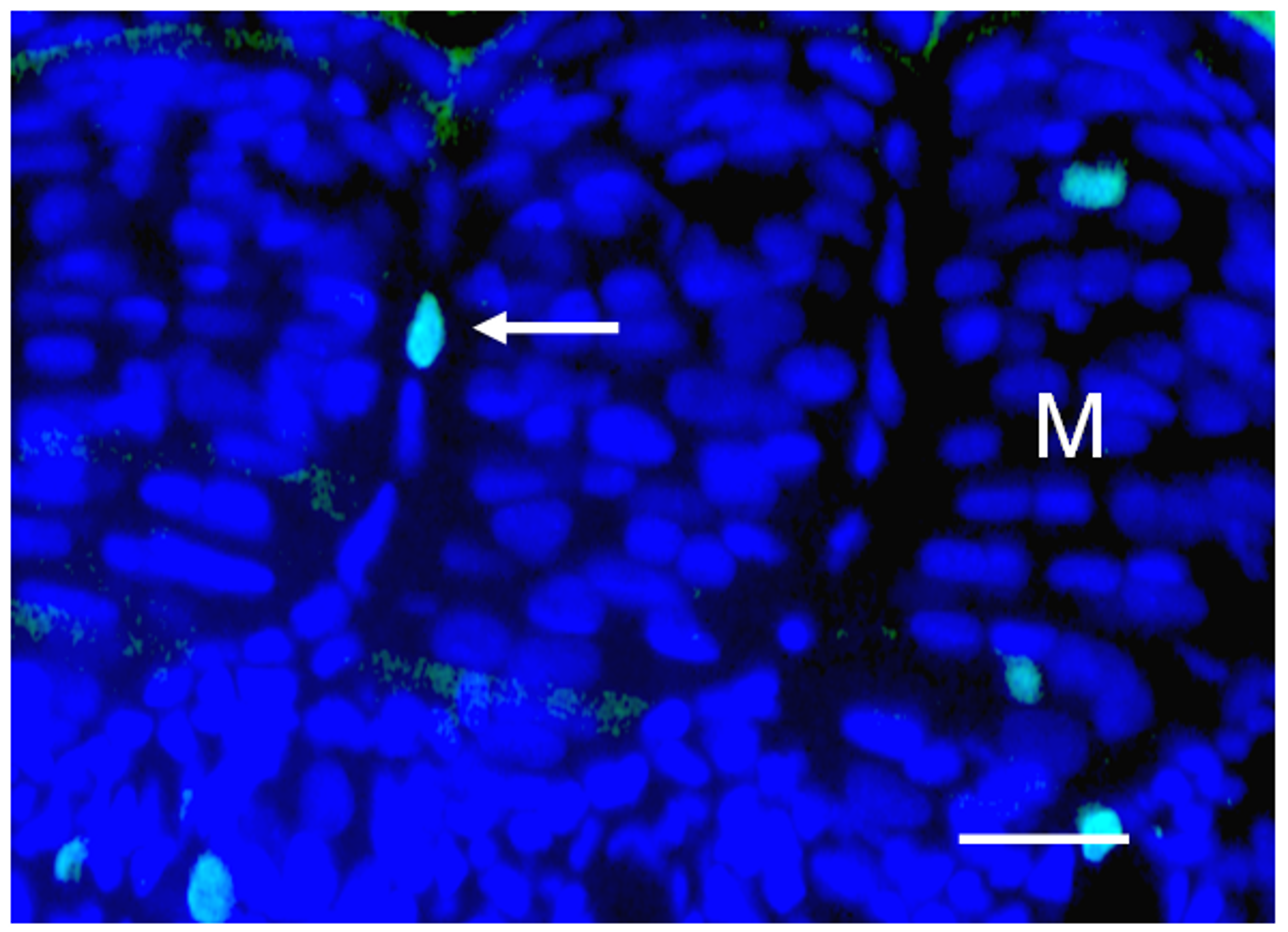

Supplement: Figure S2 — Immunolocalisation of H3P in myoseptal cells in a frontal section through the trunk of a 21 dpf trout embryo. Nuclei were stained with Hoechst. Labelling is observed in rare myoseptal cells (arrow). Scale bars, 30 μm. (TIF) [file pone.0091876.s002.tif]

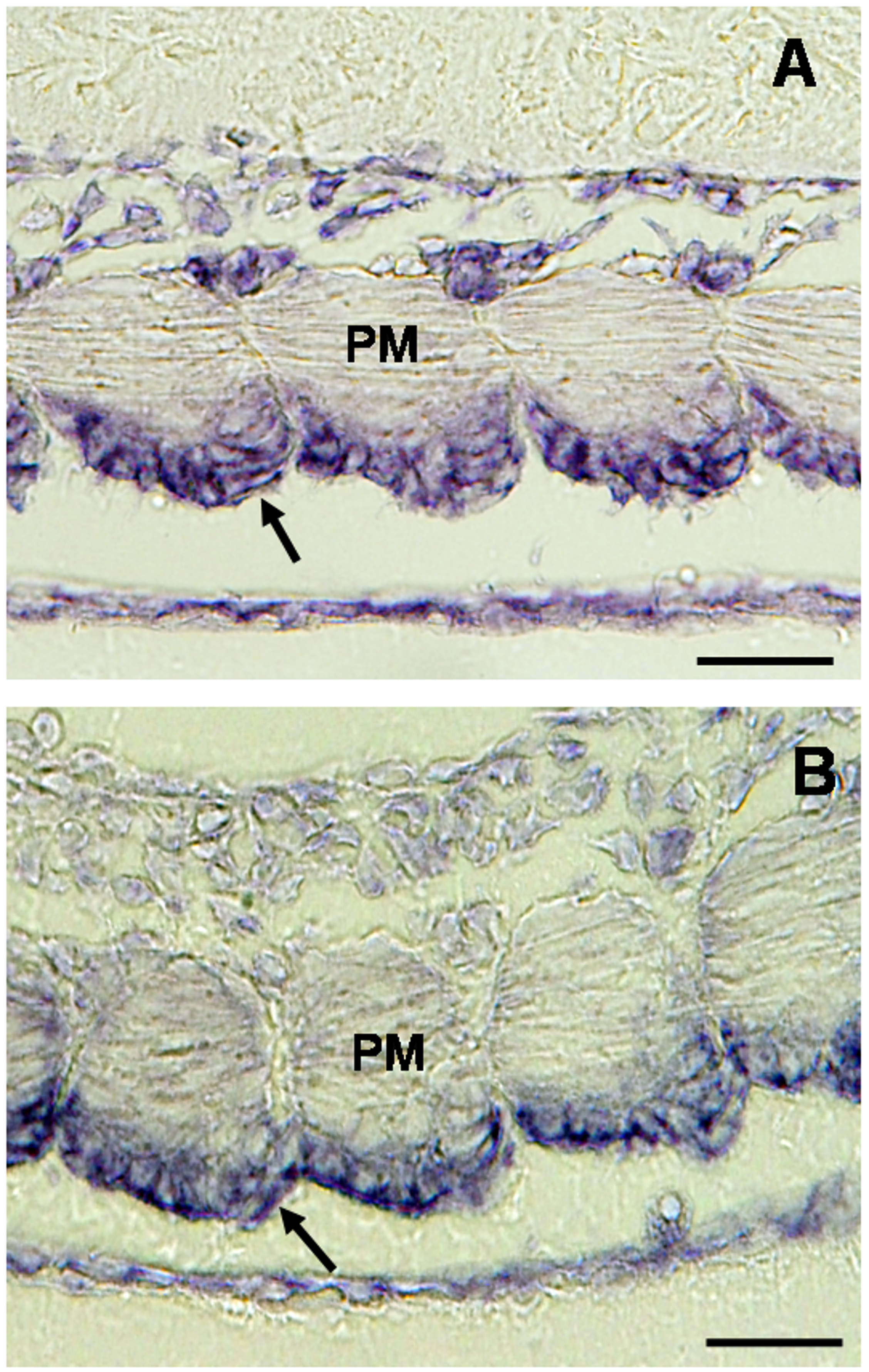

Supplement: Figure S4 — Expression of col5a2 (A) and col12a1 (B) in a trout embryo at stage 13 dpf (somitogenesis is not completed). Frontal sections. Collagen V and XII transcripts strongly accumulate in the dermomyotome-like epithelium (arrow) at the surface of the primary myotome (PM). Note that labelling for col12a1 is still reduced in sclerotome-derived cells surrounding the notochord. Scale bars in A and B, 30 μm. (TIF) [file pone.0091876.s004.tif]

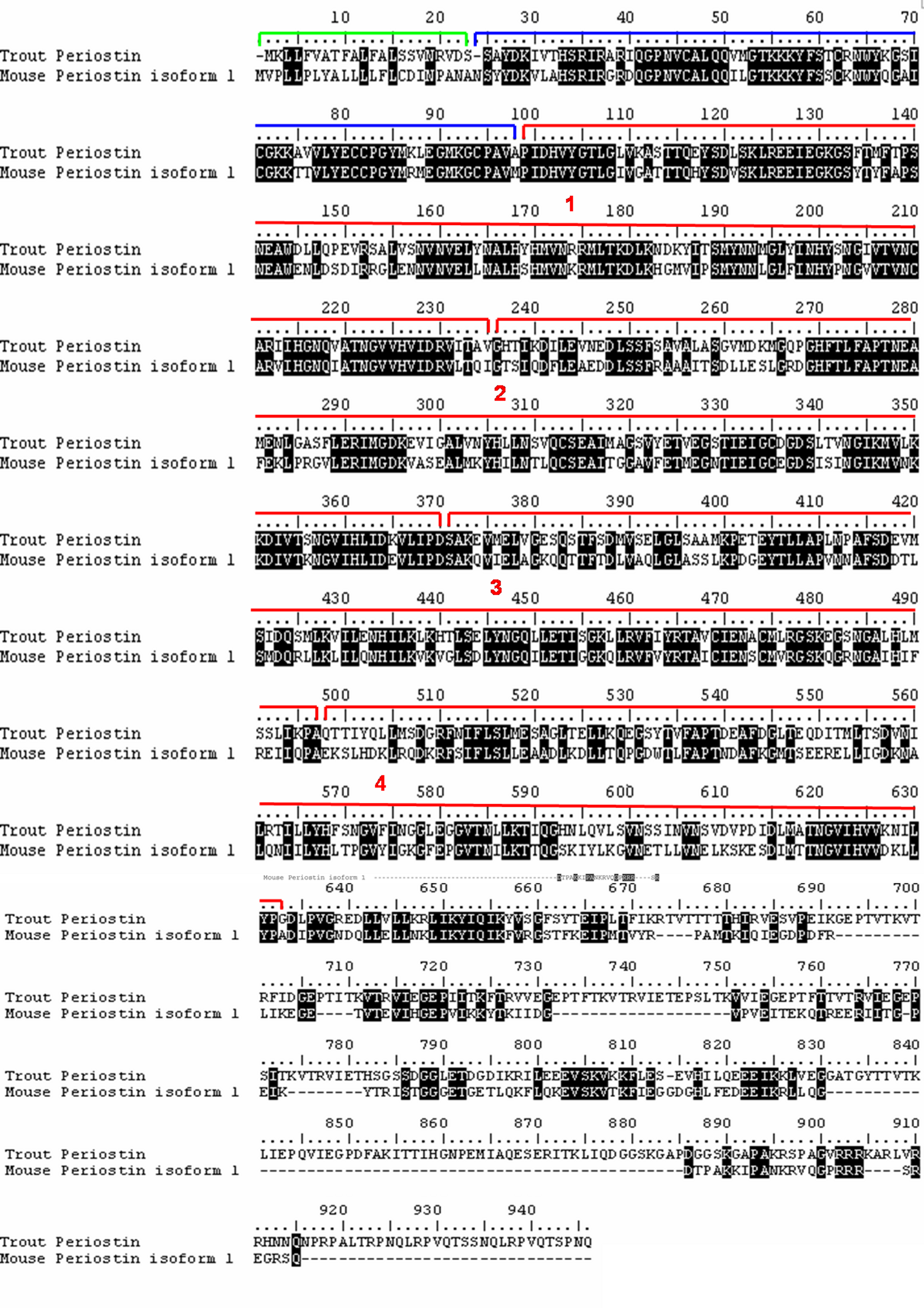

Supplement: Figure S6 — Comparison of the predicted trout and murine Osteoblast-specific factor2/Periostin protein sequence. Shading indicates identity. Positions of the signal sequence (green segment), the cysteine-rich region (blue segment) and the four fasciclin I-like repeats (R1-4) (red segments) are indicated. (TIF) [file pone.0091876.s006.tif]
